# 111. A Growing Threat: The Rising Incidence of Extended-Spectrum Beta-Lactamase-Producing Enterobacterales Infections Among Pediatric Patients in Monroe County, NY

**DOI:** 10.1093/ofid/ofaf695.043

**Published:** 2026-01-11

**Authors:** Hsioa Che Looi, Julia Tellerman, Christina B Felsen, Elizabeth L Anderson, Jenna Dietz, christopher J Myers, Rebecca Tsay, Brenda L Tesini, Ghinwa Dumyati

**Affiliations:** New York Rochester Emerging Infections Program at the University of Rochester Medical Center, Rochester, New York, Rochester, NY; New York Emerging Infections Program, Rochester, New York; University of Rochester School of Medicine and Dentistry, Rochester, NY; University of Rochester, Rochester, New York; Center for Community Health and Prevention, Rochester, New York; University of Rochester, Rochester, New York; New York Rochester Emerging Infections Program at the University of Rochester Medical Center, Rochester, New York; University of Rochester, Rochester, New York; New York Emerging Infections Program and University of Rochester Medical Center, Rochester, New York

## Abstract

**Background:**

Extended-spectrum beta-lactamase-producing Enterobacterales (ESBL-E) infections are increasing globally. Studies specific to the pediatric population in the U.S. are limited. This study aims to describe the epidemiology of ESBL-E infections in infants and children in Monroe County, NY.

**Table 1:**
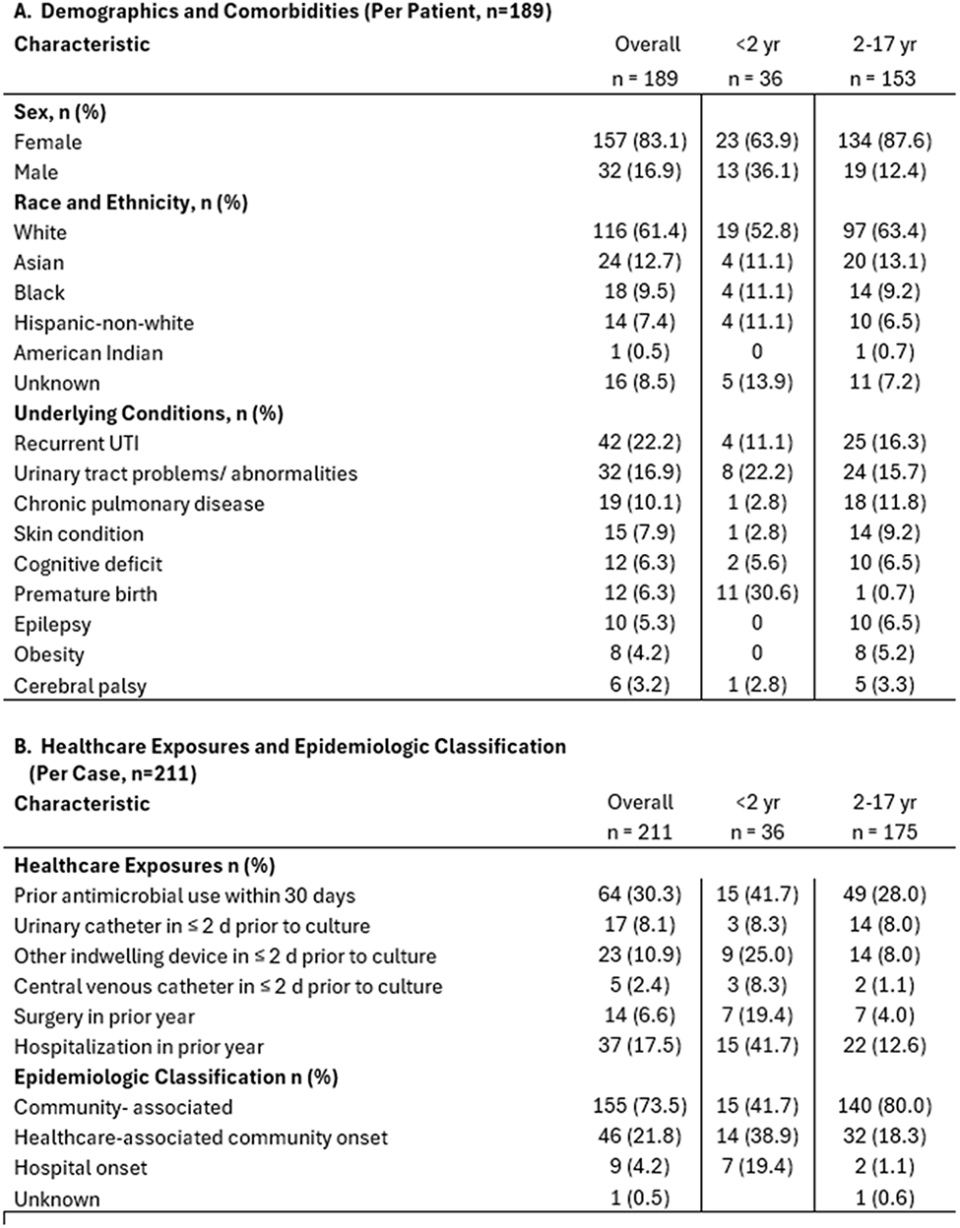
Demographic and clinical characteristics of pediatric patients with ESBL-E infections, Monroe County, July 2019-2024

**Figure 1:**
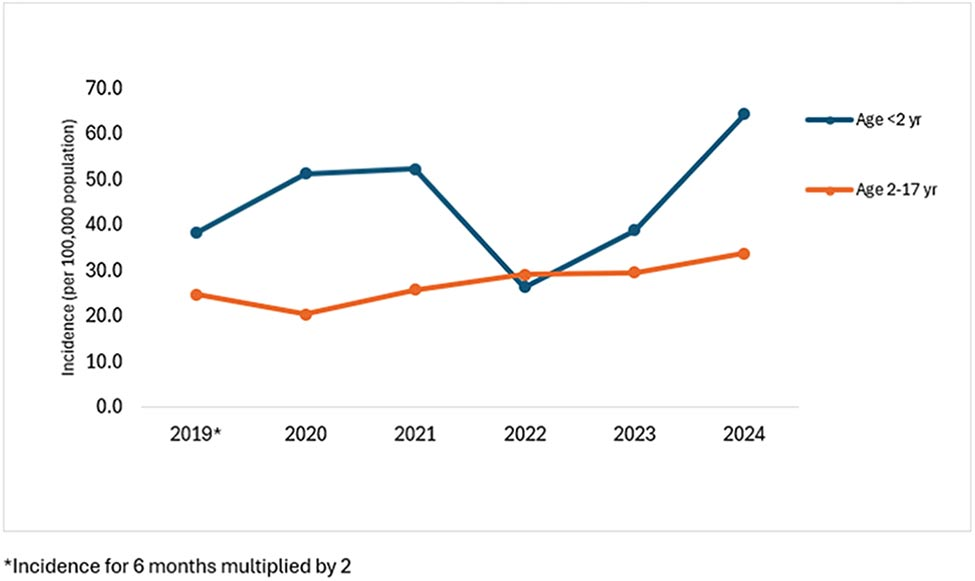
ESBL-E Incidence by age group, Monroe County, 2019-2024

**Methods:**

ESBL-E infections in residents < 18 years were identified from 7/2019 to 12/2024 through CDC Emerging Infections Program surveillance. Incident cases were defined by the first isolation of *E. coli* or *Klebsiella spp.* from sterile sites or urine within 30-day period, resistant to ≥1 3rd-generation cephalosporin but not carbapenems. Demographic and clinical data were collected for incident cases but limited to the first per year for a urine source. Cases were categorized as infants (< 2 yrs) or children (2-17 yrs). Epidemiologic classification was based on healthcare exposure; cases without such exposure were defined as community-associated (CA). Descriptive analyses were conducted using Chi-square or Fisher exact tests.

**Results:**

From July 2019-2024, we identified 247 ESBL-E cases [TB1] in 189 children. ESBL-E incidence increased from 26.1/ 100,000 population in 2019 to 36.9 in 2024. The incidence in infants rose by 68.1% over this period (Figure 1). Of the 211 cases with complete medical record review, the majority (73.5%) were CA. The epidemiologic classification varied by age group, with only 41.7% of the cases in infants classified as CA. Infants were more likely to have been born prematurely (30.6% vs. 0.7%), had a central venous catheter in place around the time of culture (8.3% vs. 1.1%), and had a hospitalization (41.7% vs. 12.6%) or surgery (19.4% vs. 4%) in the year preceding their infection. Antimicrobial use in the prior month was documented in 41.7% of infants and 28% of children. Underlying conditions were more commonly documented in infants (52.8%) than in children (38.9%). Among all pediatric patients, recurrent UTIs and urinary tract abnormalities were the most frequently observed conditions. (Table 1).

**Conclusion:**

ESBL-E incidence is rising in the pediatric population, with infants experiencing the most significant increase. Understanding age-related differences in epidemiology and risk factors is necessary to guide prevention efforts.

**Disclosures:**

All Authors: No reported disclosures

